# Atypical Presentation of Pediatric Cat Scratch Disease: A Case Report

**DOI:** 10.7759/cureus.109874

**Published:** 2026-05-29

**Authors:** Paul Bonilla, Kassandra Pulido, Natasha Bell, Otto Velasquez

**Affiliations:** 1 Medicine, University of Texas Rio Grande Valley School of Medicine, Edinburg, USA; 2 Pediatrics, University of Texas Rio Grande Valley School of Medicine, Edinburg, USA; 3 Pediatrics, Driscoll Children’s Hospital, Edinburg, USA

**Keywords:** cat scratch disease, endocrinology and diabetes, infection immunology, pediatric case, pediatric infectious disease

## Abstract

Cat scratch disease is a zoonotic infection caused by *Bartonella henselae* and typically presents with regional lymphadenopathy and mild systemic symptoms in immunocompetent children. Atypical disseminated disease with visceral involvement is uncommon and may occur without classic clinical findings, leading to delayed diagnosis, particularly in patients with altered immune responses. We describe the case of an 11-year-old girl with type 1 diabetes mellitus who presented with persistent fever and progressive abdominal pain. Initial evaluation suggested streptococcal pharyngitis, and she was treated with appropriate antibiotics without improvement. Subsequent imaging revealed multiple hepatic lesions concerning for malignancy or infection. The patient had significant cat exposure but lacked lymphadenopathy or cutaneous lesions. Laboratory studies demonstrated anemia and elevated inflammatory markers with normal liver enzymes. Magnetic resonance imaging confirmed multifocal hepatic involvement. Based on imaging findings and exposure history, disseminated *Bartonella henselae* infection was suspected, and empiric antimicrobial therapy was initiated. The patient improved clinically, and serologic testing later confirmed the diagnosis. This case highlights an atypical presentation of disseminated cat scratch disease with isolated hepatic involvement in a child with diabetes mellitus. Recognition of hepatic lesions on imaging and careful exposure history were critical for diagnosis. Clinicians should maintain a high index of suspicion for *Bartonella henselae* in pediatric patients with prolonged fever and abdominal pain, especially those with metabolic or immunologic risk factors, even in the absence of classic features.

## Introduction

*Bartonella henselae *is commonly* *transmitted through scratches or bites from cats, particularly kittens. Historically, diagnosis relied on clinical criteria that included a history of animal contact, the presence of an inoculation site, regional lymphadenopathy, and positive skin testing [1]. This classic presentation of a papule or pustule at the inoculation site is often followed by regional lymphadenopathy and low-grade fever [1]. In most immunocompetent pediatric patients, the disease is self-limited and resolves without complication [2].

Atypical and disseminated forms of infection have been increasingly recognized, particularly in children with altered immune responses. Hepatic and splenic lesions occur in a small subset of patients and may present without the hallmark findings of lymphadenopathy or cutaneous lesions, complicating diagnosis [3,4]. This report describes an atypical presentation of disseminated *Bartonella henselae* infection in an 11-year-old girl with diabetes mellitus, highlighting the diagnostic importance of hepatic lesions and the need for heightened clinical suspicion in high-risk pediatric populations.

## Case presentation

An 11-year-old girl with a history of poorly controlled type 1 diabetes mellitus (HbA1c: 8.4%) presented to her primary care pediatrician with fever and diffuse abdominal pain. She weighed approximately 36 kg and was managed on a basal-bolus insulin regimen consisting of insulin glargine 13 units subcutaneously once daily at bedtime, and insulin lispro 4 units subcutaneously three times daily with meals, with a correction factor of 1 unit per 55 mg/dL above a target glucose of 120 mg/dL.

Her symptoms began two days prior and were associated with malaise and decreased appetite. On examination at the pediatrician’s office, she was febrile, and oropharyngeal erythema was noted. Given the presence of fever, sore throat symptoms, and the local prevalence of streptococcal pharyngitis, a rapid antigen detection test for group A *Streptococcus* was performed and returned positive. She was prescribed oral amoxicillin and discharged home.

Despite adherence to antibiotics, her symptoms persisted. She continued to have daily fevers with a maximum temperature of 102°F (38.8°C), chills, body aches, and worsening abdominal pain. On day three of illness, she presented to the emergency department. Repeat evaluation included a second rapid streptococcal test and confirmatory throat culture, both of which were positive, reinforcing the initial diagnosis. She was advised to continue the prescribed antibiotic regimen.

On day four, the patient returned to the emergency department due to worsening right upper quadrant abdominal pain. At this visit, she disclosed frequent exposure to cats, including direct contact with stray cats that roamed her yard. Vital signs were notable for persistent fever and mild tachycardia. Physical examination revealed right upper quadrant abdominal tenderness without guarding or rebound. No lymphadenopathy, hepatomegaly, splenomegaly, or skin lesions were identified.

Given concern for appendicitis or other intra-abdominal pathology, computed tomography (CT) of the chest and abdomen was obtained. This was ordered to evaluate for additional sites of disease and to exclude a primary pulmonary process. Imaging revealed multiple hypodense cystic lesions within the liver, raising concern for malignancy or infectious etiologies. The patient was admitted for further evaluation.

Laboratory testing demonstrated normocytic anemia (mean corpuscular volume: 92 fL) with a hemoglobin level of 9 g/dL (reference range: 11.9-14.8 g/dL), normal platelet count, elevated erythrocyte sedimentation rate of 66 mm/hour, and elevated C-reactive protein of 7.7 mg/L. Liver function tests were within normal limits (Table 1).

**Table 1:** Summary of key laboratory findings.

Test	Result	Reference range
Hemoglobin	9.0 g/dL	11.9–14.8 g/dL
Platelet count	204 × 10^3^/µL	150–450 × 10^3^/µL
Erythrocyte sedimentation rate	66 mm/hour	<20 mm/hour
C-reactive protein	7.7 mg/L	<3 mg/L
Aspartate aminotransferase	22 U/L	15–35 U/L

Due to persistent fever, cough, and chest pain despite antibiotic therapy, a respiratory viral panel was obtained, and was positive for human coronavirus OC43 and rhinovirus.

**Figure 1:**
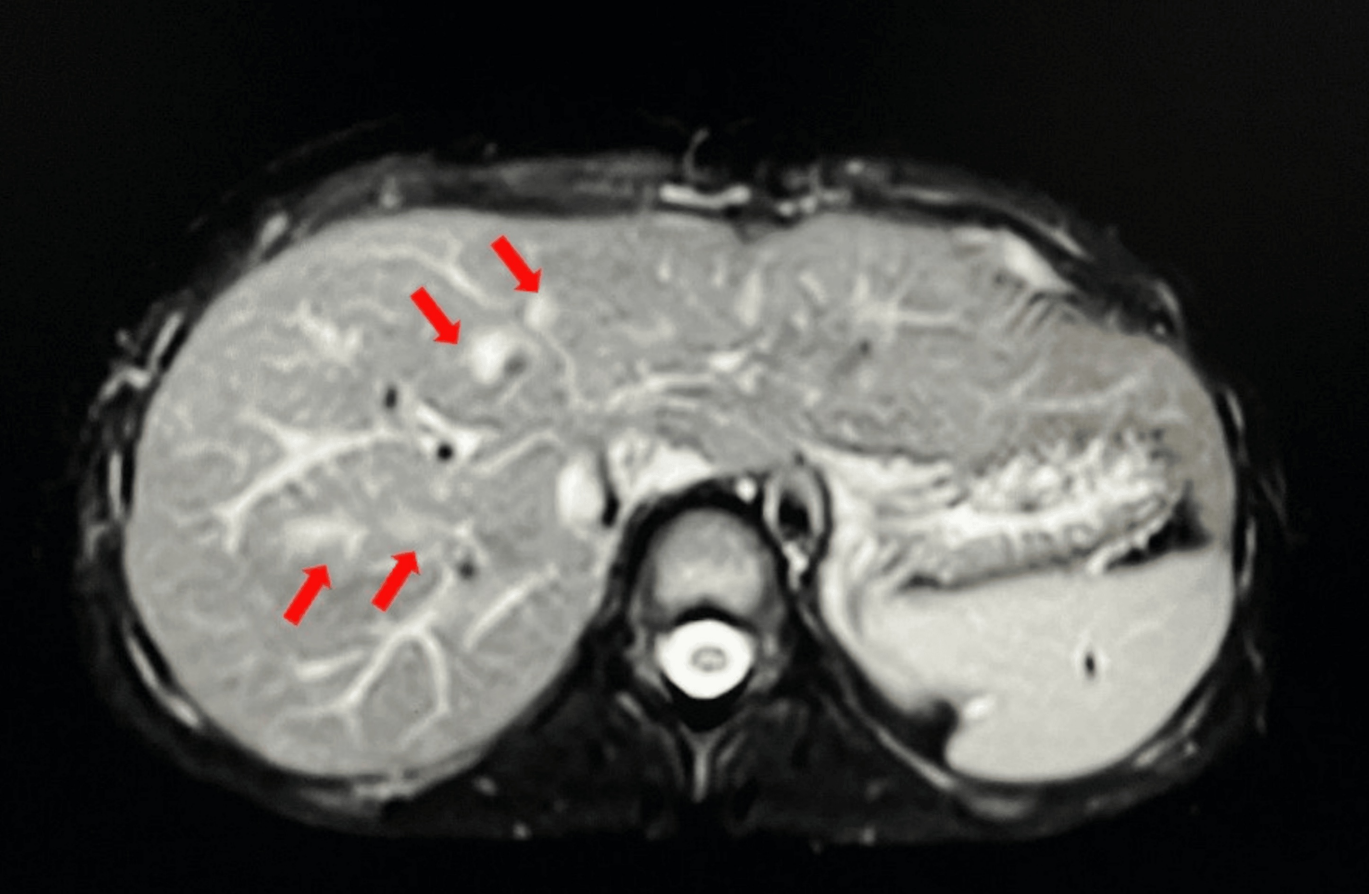
Abdominal magnetic resonance imaging showing multiple hepatic lesions.

Magnetic resonance imaging (MRI) of the abdomen further characterized the hepatic lesions, revealing multiple well-circumscribed lesions, the largest measuring 1.7 × 1.4 cm, consistent with focal hepatic involvement rather than neoplastic disease (Figure 1).

**Figure 2:**
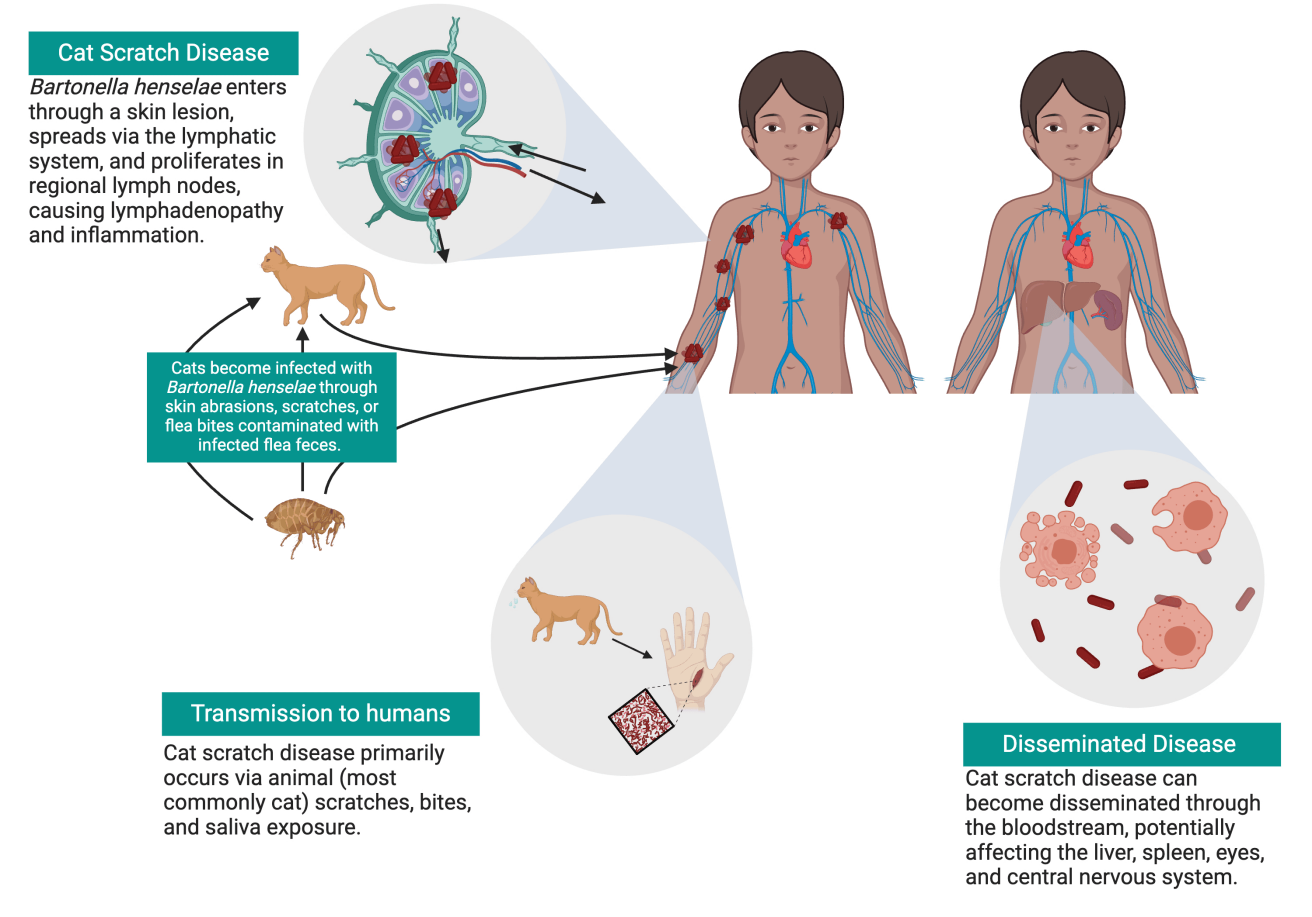
Transmission of Bartonella henselae and outcome following human infection. Created in BioRender (https://BioRender.com/7alugmk).

Given her history of cat exposure, persistent fever, and characteristic hepatic lesions, disseminated *Bartonella henselae* infection was suspected (Figure 2).

**Figure 3:**
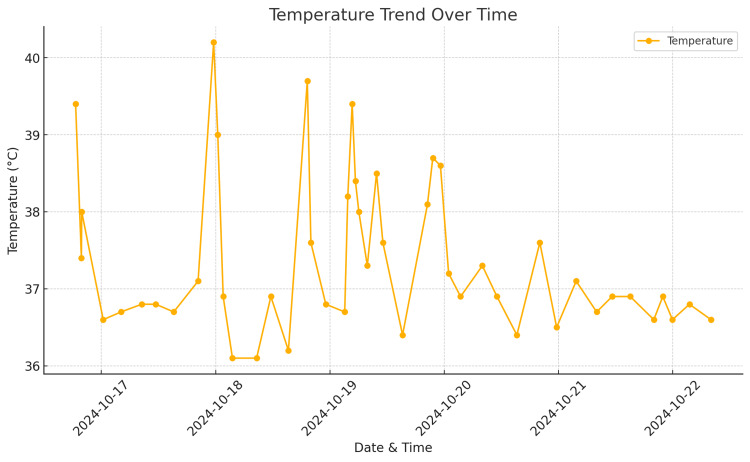
Temperature trend throughout the hospital stay.

Empiric therapy with doxycycline and intravenous gentamicin was initiated while awaiting serologic testing. The patient continued to have intermittent fevers until hospital day seven, after which she became afebrile (Figure 3). She was discharged on oral azithromycin and rifampin to complete a two-week course.

Serologic testing obtained during hospitalization later returned positive for *Bartonella henselae* immunoglobulin G, confirming the diagnosis of disseminated cat scratch disease.

## Discussion

This case illustrates an atypical presentation of cat scratch disease characterized by visceral involvement without the classic findings of lymphadenopathy or cutaneous inoculation lesions [3,4]. While most pediatric cases of cat scratch disease are localized and self-limited, disseminated disease involving the liver, spleen, eyes, or central nervous system has been reported in approximately 5-10% of cases [5].

Hepatic involvement is a recognized but uncommon manifestation of *Bartonella henselae* infection. Hepatic lesions typically appear as multiple hypodense or cystic lesions on CT or MRI and may be mistaken for malignancy, abscesses, or granulomatous disease [6]. In several published pediatric case series, the presence of hepatic or hepatosplenic lesions in the setting of prolonged fever and cat exposure was a key diagnostic clue leading to serologic confirmation of *Bartonella henselae* [7]. Thus, recognition of liver lesions plays an important diagnostic role, particularly when classic clinical features are absent.

The patient’s underlying diabetes mellitus likely contributed to the atypical and disseminated nature of her infection. Diabetes has been associated with impaired innate and adaptive immune responses, including neutrophil dysfunction, altered cytokine signaling, and intracellular killing [8]. Recognition of atypical presentations is particularly important in children with metabolic or immunologic vulnerabilities. Case reports and cohort studies suggest increased susceptibility to systemic disease and delayed diagnosis in this population.

This presentation was further complicated by concurrent streptococcal pharyngitis and viral respiratory infections, which likely contributed to diagnostic anchoring early in the clinical course. The persistence of fever despite appropriate antibiotic therapy, combined with progressive abdominal pain and imaging findings, ultimately prompted reconsideration of the diagnosis. This underscores the importance of reassessing initial assumptions when patients fail to improve as expected.

Disseminated cat scratch disease may lead to long-term complications, including chronic abdominal pain, persistent hepatosplenic lesions, neuroretinitis, encephalopathy, and, rarely, endocarditis [9]. Early recognition and targeted antimicrobial therapy are essential to prevent these outcomes. In pediatric patients with prolonged fever of unknown origin, especially those with diabetes or other risk factors, *Bartonella henselae* infection should remain part of the differential diagnosis even in the absence of lymphadenopathy.

Several limitations of this case report warrant acknowledgment. First, definitive microbiologic confirmation via polymerase chain reaction (PCR) was not performed. Although serology remains the most widely used diagnostic modality for *Bartonella henselae* in clinical practice, PCR of blood or tissue provides direct pathogen detection and would have strengthened the diagnostic certainty of this case. Second, histopathologic confirmation through liver biopsy was not obtained. Biopsy of hepatic lesions can yield characteristic granulomatous inflammation and, when combined with Warthin-Starry silver staining or PCR, can provide a definitive tissue-based diagnosis. The decision to forgo biopsy was made in the context of clinical improvement with empiric therapy; however, the absence of histopathologic data limits the ability to fully exclude alternative etiologies such as fungal infection or malignancy. Third, follow-up data were limited, precluding assessment of long-term outcomes, including resolution of hepatic lesions, recurrence of infection, or the development of delayed complications such as neuroretinitis or chronic abdominal pain. Future case series with longer follow-up periods and comprehensive microbiologic workup would better characterize the natural history of disseminated cat scratch disease in pediatric patients with diabetes mellitus.

## Conclusions

This case highlights an atypical presentation of pediatric cat scratch disease characterized by hepatic involvement and prolonged fever in a child with diabetes mellitus. The absence of classic clinical findings delayed diagnosis, emphasizing the importance of maintaining a broad differential in children with persistent fever and abdominal pain. Recognition of hepatic lesions on imaging, combined with a careful exposure history, was critical in identifying disseminated *Bartonella henselae* infection. Clinicians should maintain heightened suspicion for atypical presentations in patients with metabolic or immunologic vulnerabilities, particularly in regions with high rates of diabetes, to facilitate timely diagnosis and prevent long-term complications.
